# Blood Cells Separation and Sorting Techniques of Passive Microfluidic Devices: From Fabrication to Applications

**DOI:** 10.3390/mi10090593

**Published:** 2019-09-10

**Authors:** Susana O. Catarino, Raquel O. Rodrigues, Diana Pinho, João M. Miranda, Graça Minas, Rui Lima

**Affiliations:** 1 Center for MicroElectromechanical Systems (CMEMS-UMinho), University of Minho, Campus de Azurém, 4800-058 Guimarães, Portugal; 2 Research Centre in Digitalization and Intelligent Robotics (CeDRI), Instituto Politécnico de Bragança, Campus de Santa Apolónia, 5300-253 Bragança, Portugal; 3 CEFT, Faculdade de Engenharia da Universidade do Porto (FEUP), Rua Roberto Frias, 4200-465 Porto, Portugal; 4 MEtRICs, Mechanical Engineering Department, University of Minho, Campus de Azurém, 4800-058 Guimarães, Portugal

**Keywords:** microfluidics, red blood cells (RBCs), microfabrication, polymers, separation and sorting techniques

## Abstract

Since the first microfluidic device was developed more than three decades ago, microfluidics is seen as a technology that exhibits unique features to provide a significant change in the way that modern biology is performed. Blood and blood cells are recognized as important biomarkers of many diseases. Taken advantage of microfluidics assets, changes on blood cell physicochemical properties can be used for fast and accurate clinical diagnosis. In this review, an overview of the microfabrication techniques is given, especially for biomedical applications, as well as a synopsis of some design considerations regarding microfluidic devices. The blood cells separation and sorting techniques were also reviewed, highlighting the main achievements and breakthroughs in the last decades.

## 1. Introduction

Since the development of the first microfluidic device, microfluidics heralded the promise to change life science and industry [1]. Despite the enormous scientific achievements that microfluidics have had in the last decades in the field of biomedical applications, this technology is still considered in its “adolescence” [2]. Among the pullbacks, are the difficulty to achieve a cost-effective large-scale production that allows its commercialization for clinical application, the so-called lab-on-a-chip, and the complete understanding of the physics of fluids at the microscale level over the biological species, such as blood and blood cells.

Blood and blood cells are important for scientific and clinical purposes because they can be used as indicators of many pathological conditions, including arterial hypertension, ischemia, inflammation, and diabetes [3,4]. Based on the fact that abnormal blood cells typically have distinctive biological and physicochemical properties (e.g., size, deformability and chemical composition), with different hydrodynamic properties when compared to healthy ones, these features can be used for rapid, low-cost cell separation and diagnosis.

In parallel, recent developments in microfabrication with polymers and elastomers made possible to fabricate low-cost transparent micrometre-sized channels and, as a result, several studies have been proposed using microfluidics to measure the motion and dynamic behaviour of cells flowing through microfluidic devices [5,6,7,8,9,10,11,12]. Taken into consideration that since its origins, microfluidics has flourished and paved its path in parallel with the development of new fabrication technologies, the present review aims to give an overview perspective of this technology for blood cells separation and sorting from the fabrication to application, and thus, revising the main achievements and breakthroughs in the last decades.

This review is organized as follows: Section 2 presents a description of different techniques for the fabrication of microfluidic devices, as well as a comparison between them; Section 3 approaches design considerations regarding microfluidic devices for biomedical applications; Section 4 describes and compares the main passive methods for cells sorting and separation; and Section 5 briefly discusses future challenges and perspectives regarding microfluidic devices and their applications.

## 2. Fabrication of Polymeric Microfluidic Devices

**Figure 1:**
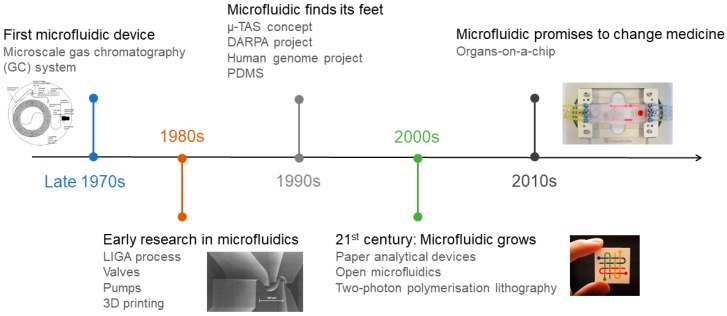
Timeline of the main microfluidics achievements from the first microfluidic device until the present.

The beginning of microfluidics and its early systems, in the late 1970s (Figure 1), were derived from microelectronics and microelectromechanical systems (MEMS) technology and techniques, such as photolithography and etching, which were highly developed at the time [2,13].

**Figure 2:**
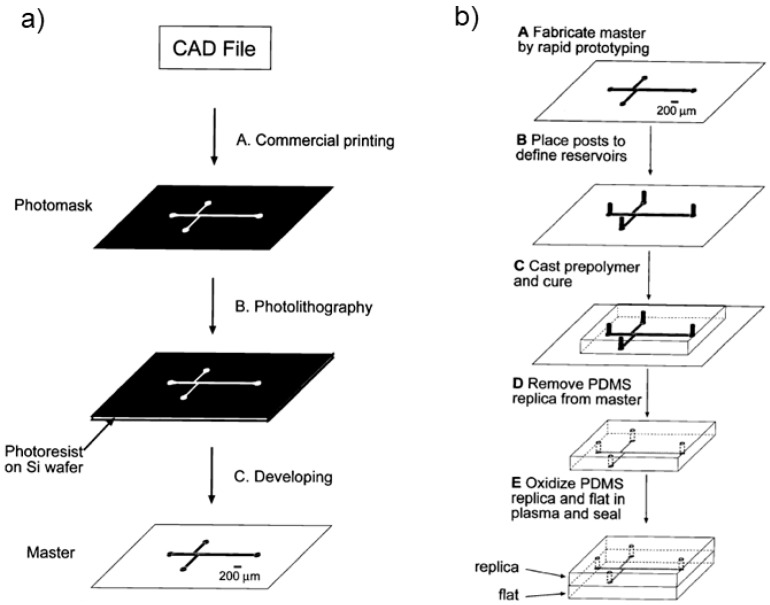
Soft lithography technique introduced by Whitesides and co-workers in 1998. (**a**) Rapid prototyping using photolithography and (**b**) replica molding with poly(dimethylsiloxane) (PDMS). Reproduced with permission from [13].

Initially, silicon and glass were the select material to produce those microfluidic devices. Although, silicon had a big impact in microelectronics, initiating the Silicon Valley revolution, the material has some disadvantages for microfluidics, such as its opacity in the Ultra-Violet/Visible (UV/Vis) region of the electromagnetic spectrum and relative high cost [13,14]. Glass, on the other hand, is transparent, but due to its amorphous structure is difficult to etch compared with pure SiO_2_. For the pattern of small size structures, sandblast and wet etching are the most used techniques. Nevertheless, sandblast is typically limited for patterns below 100 m and leads to rough surfaces, while wet etching allows smooth sidewalls but has low aspect ratio [15]. Therefore, among the glass micromachining limitations are the low etching aspect ratio and rate, limited mask selectivity and surface roughness [15]. Other disadvantages are that both materials required that each device is made in cleanroom facilities and its sealing made with high voltages and temperatures, which makes the microfabrication laborious and expensive [13]. In contrast to glass and silicon, polymers and elastomers, are less expensive and the channels can be obtained by molding or embossing that makes the fabrication faster and less expensive [14]. Among the most popular polymers used to fabricate microfluidic devices are poly(methyl methacrylate) (PMMA), cyclic olefin copolymer (COC), poly(styrene) (PS), poly(carbonate) (PC), poly(ethyleneterephthalate glycol) (PETG) and poly(dimethylsiloxane) (PDMS) [14]. Derived from this effort to find alternative materials, PDMS, a transparent elastomeric polymer pioneered by George Whitesides and his group at Harvard in the 1990s, quickly become the most popular material used in microfluidic devices [2]. The use of this new material made possible the massification of soft-lithography technique, with rapid prototyping and replica molding (Figure 2).

Figure 2 shows the main steps involved in the design and fabrication of microfluidic devices using the soft-lithography technique. The detailed description of these steps is well described elsewhere [13].

This innovation allowed the growth of microfluidics field due to the many advantages of this material: (i) high fidelity to replicate by molding features at the micro-scale level; (ii) its optically transparent down to 280 nm; (iii) low temperature and time to cure; (iv) biocompatibility and nontoxicity to cells; (v) possibility to change surface chemistry accordingly to the application needs; (vi) gas permeability, allowing culture of cells; (vii) reversal and self-bonding, among others [13,16,17].

In general, soft-lithography follows four major steps: (i) pattern design, drawn in computer-aided design (CAD) software programs for the fabrication of photomasks on transparency films (Figure 2a); (ii) fabrication of the mask and master, photomasks on transparency films are designed in high-resolution printers followed by photolithography technique (Figure 2a); (iii) fabrication of the PDMS stamp, fabricated by casting PDMS (pre-polymer mixed with cure agent) against a master whose surface has been patterned (Figure 2b) and (iv) fabrication of micro- and nanostructures with the stamp by printing, molding and embossing [18].

Although the several advantages of the soft-lithography method with PDMS, the standard prototyping method (i.e., photolithography) requires the access to cleanroom facilities and high-trained people. Additionally, the replica molding with PDMS is achieved by casting the masters one by one, which make the large-scale production slow. Nevertheless, this process is an ideal and fast solution to test prototypes. Another important aspect is that despite the common statement that BioMEMS is straightforward and inexpensive, the fabrication of microfluidic devices is, in general, complex and costly. For instance, it is estimated that the user fee in the United States for a fully staffed cleanroom, in a major research university, is in the order of $100/h per student [19]. This must include the typical time for training that can take several weeks for the basic operation of equipment and familiarization of techniques, such as spin coater, masker aligner and developing station [19]. An alternative is the contract of manufacturers that can provide custom master molds for a relatively low fee. However, this process can take several weeks from manufacturing to shipping [20]. To suppress the high cost and constrains of photolithography, alternatives have been developed in the last decade for the low-cost of microstructures without the need of cleanroom facilities. An example of this effort was published by Pinto et al., 2014 [21], describing the fabrication and optimization of microstructures in SU-8 (commonly used as epoxy-based negative photoresist), without the need of cleanroom facilities. The proposed fabrication technique uses an alternative photomask printed in transparent photographic sheet using standard tools and equipment employed in the printed circuit board (PCB) industry. Even though the outstanding achievement of the proposed technique, the SU-8 shows a resolution limitation of 10 m and the need to control the room temperature and humidity to optimize the fabrication procedure.

**Figure 3:**
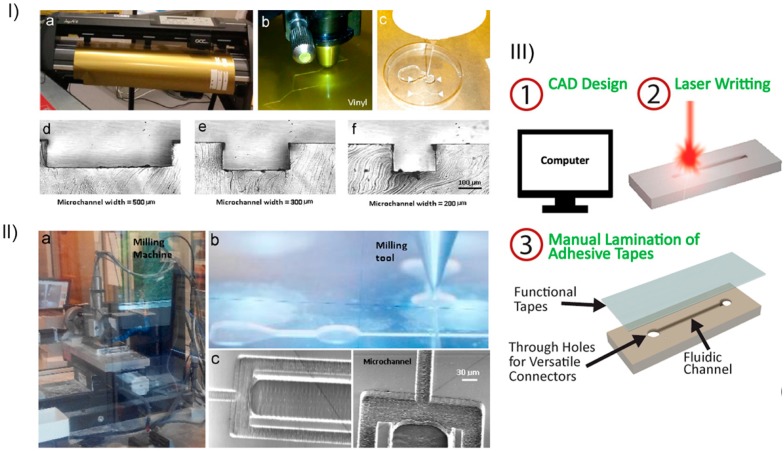
Low-cost print-and-peel microfabrication techniques. (**I**) Xurography: (**a**) cutting plotter machine; (**b**) features being cut by the cutting plotter; (**c**) PDMS being added to a petri dish containing the vinyl mask; (**d**), (**e**) and (**f**) Cross sections of microchannels with 500, 300 and 200 m of width, respectively. (**II**) Micromilling; (**a**) milling machine; (**b**) operating milling tool and (**c**) microchannels. Reproduced with permission from [22]. (**III**) Direct laser plotting main steps. Reproduced with permission from [27].

In parallel, alternative non-lithographic techniques have also emerged from these requirements of specific facilities and equipment that has inhibited many scientific groups to pursue new microfluidic innovations, namely print and peel techniques, e.g., xurography, micromilling or direct laser plotting (Figure 3), which are well reviewed elsewhere [22].

Briefly, xurography allows the generation of master molds (or masks) using a cutting plotter machine and adhesive vinyl film. Recently, Pinto et al. (2014) [23] have shown that xurography can be used as a rapid technique with good resolution to produce microfluidic structures down to 500 m. By using this technique Bento et al. [24] were able to successfully produce microchannels contractions with dimensions down to 350 m and as a result they have investigated how Taylor bubbles disturb the blood flow at the scale of blood cells.

Micromilling, is another low-cost fabrication technique that creates microscale structures by removing bulk material with cutting tools [25]. This technique was shown by Lopes and co-workers to have the ability to produce reusable microfluidic devices with widths down to 30 m [26].

Direct laser plotting is a microfabrication technique similar to micromilling that uses laser beams to create microchannels. This technique can typically generate microchannels widths up to 100 m. Although it has been shown the possibility to down to 20 m by using short laser pulses [22].

3D-printing fabrication techniques have also gained a growing interest to fabricate microfluidic devices, offering the possibility to generate devices with complex architectures from a broader range of materials and avoiding multi-step processing [28]. The main 3D-printing techniques are stereolithography (SLA), fused deposition modelling (FDM), selective laser sintering (SLS) and direct ink writing (inkjet) [28,29,30], allowing a broad range of applications. Among the advantages, the simplicity, fast and efficient prototyping with no need of photomask and cleanroom facilities, are some of the most important.

FDM, is the most simple and low-cost 3D-printing method, working by extruding a thermoplastic polymer through a hot nozzle to print layers of the object. The technique can be used to produce directly the microfluidic devices or the 3D mask that combined with PDMS replication molding allows the fabrication of 3D-biomodels, such as macro and micro-scale vascular system models [30,31].

An overview of the main advantages and disadvantages of the most representative fabrication techniques used to develop microfluidic devices in polymer substrates is given in Table 1.

With the recent development of nanotechnology for several applications and fields, nanofabrication techniques for microfluidic devices have also been developed. In general, these new techniques are based in advanced nanoscale photolithographic methods, such as extreme ultraviolet, electron beam and nanoimprint lithography, or non-lithographic methods, such as anodic aluminium oxidation. All these new nanofabrication approaches are well described elsewhere [17]. With the ability to generate features with just a few nanometres, the main application of these nanofabrication techniques are lab-on-a-chip microdevices, with high potentiality to medicine, biology and chemical applications [47,48,49].

## 3. Design of Microfluidic Devices for Biomedical Applications

Biomedical science found a fruitful field in microfluidics to replace routine analysis and diagnosis tests, as well as to conduct fundamental biological studies in cells and diseases. Among the biomedical applications, microfluidics research has allowed the emerging of a wide range of promising applications from microscale genomic and proteomic analysis kits, biosensors, point-of-care diagnostic devices, drug screening and delivery platforms, implantable devices, novel biomaterials to tissue engineering and single cell studies [50].

**Figure 4:**
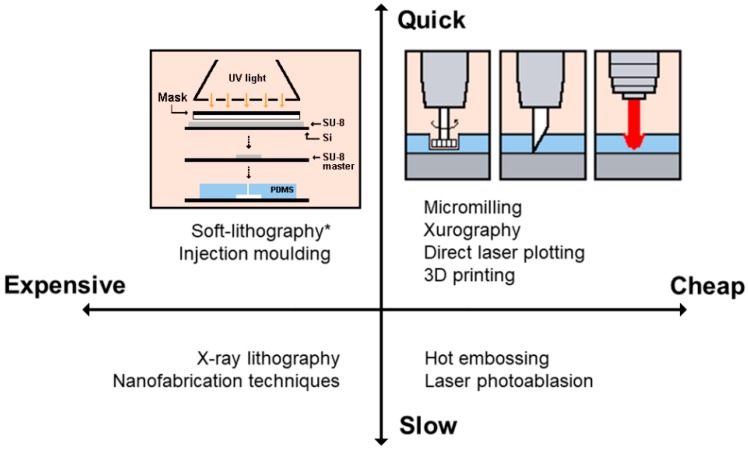
Fabrication techniques from a time and cost perspective. Adapted from [14]. ^*^ Despite standard soft-lithography technique is considered expensive, new alternatives without the need of cleanroom facilities significantly drop the cost, being considered as low-cost, as the work published by Pinto et al., 2014 [21].

Depending on the final application of the microfluidic device, different micro- or nanofabrication techniques are available and can be used. In general, most of the research groups try to pursue a time-cost effectiveness to fabricate their own microdevices. Based on this standpoint, Figure 4 gives an overview of the fabrication techniques listed in Table 1, from a time and cost perspective.

The material selection also has an important role in the application. For biomedical applications the selection of the material must consider important parameters, namely biocompatibility, bio-culture, permeability and porosity, protein crystallization, reusability and disposable device use. Some of these characteristics are listed in Table 2, for the most common materials used for biomedical applications.

Another important aspect for the fabrication of microfluidic devices is the interfacing and/or integration of modules for the applications that the device is being designed. Among them, integration of microheaters, valves, sensors, electroosmotic fluid pumps, readout electronics, among others, can be accomplished to complete microfluidic devices with remarkable capabilities [52].

## 4. Microfluidic Cell Separation and Sorting Techniques

Despite all the research and development of microfluidic systems, several challenges remain related to the miniaturization of the lab-on-a-chip devices. At the microscale level, the mixture, pumping, separation and control of fluids are limited, on the one hand, by the minimum sample volumes and flow rates required by the biological analysis and, on the other hand, by the microscale dimensions of the systems. The dominant physical and chemical effects at the microscale level are different from the ones at the macroscale, leading to an increased complexity of the flow and mass transport phenomena. In order to overcome those limitations, significant research efforts have been performed for improving the design of micropumps, valves, mixers and separation devices that can be incorporated on lab-on-a-chip devices [53,54,55], while addressing the non-Newtonian behaviour of the majority of physiological fluids [56,57].

Microfluidic systems can integrate different kinds of sorting methods based on the physical parameters of cells, providing a perfect interface for the manipulation of single cells and access forces in a variety of ways and allowing a fully autonomous measurement of physical parameters [58]. Particularly, cell separation techniques have been developed for cell concentration purposes (removal of plasma and increase of the cell concentration, mainly haematocrit increase); plasma enrichment (removal of cells from plasma and cells dilution); blood fractioning (separation of blood into different components); cell sorting (separation of cells by type); and cell removal (specific cell sorting that removes only some specific cells), that can work as cell isolation or removal of pathogenics [59].

**Figure 5:**
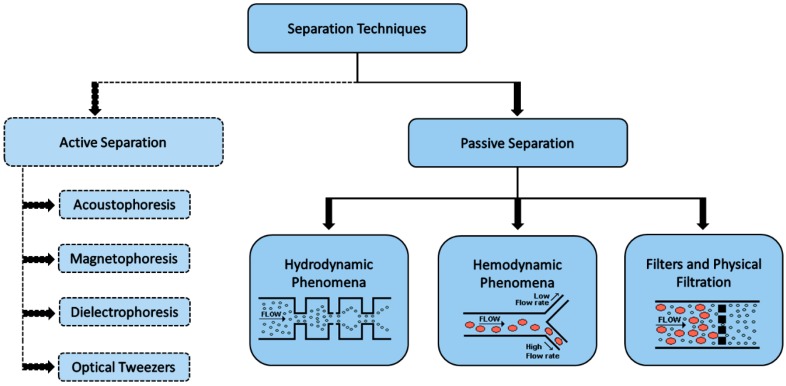
Classification of the main active and passive separation techniques used in microfluidic systems.

The manipulating of forces for the separation techniques can be active, passive or both (label-free cell sorting mechanism), as shown in Figure 5. Apart from these, there are other methods such as paper-based [60,61] and CD based [62,63] methods to separate mainly the plasma from blood [64]. Active technologies, based on microelectromechanical systems, improve the control of fluids using mobile parts or external mechanical forces, and can be based on dielectrophoresis, magnetophoresis, acoustophoresis and optical tweezers mechanisms [58,64]. Passive technologies for controlling fluids do not include external forces or mobile parts, and their control is promoted by diffusion as a function of the channel geometry [64,65,66,67,68,69,70], or intrinsic hydrodynamic forces, such as punch flow fraction, deterministic lateral displacement, inertial forces and intrinsic physical property of the cells [69,70,71,72,73,74], including sieving, which uses the size of micropores, microweirs, membranes and the gap between micropillars arrays for the separation of cells [26,69,70,71,72,73,74,75,76,77,78]. The passive microfluidic technologies bring more interest in the lab-on-a-chip and microfluidics research field due to its precise manipulation, low cost fabrication, simple structure, simple integration and lower maintenance in lab-on-a-chip devices and high throughput [79,80,81,82].

Therefore, this paper presents, in addition to an overview over the microfabrication techniques using polymers as substrates, a review and discussion of different passive techniques and microfluidic devices for separation of cells, categorized according to the separation phenomena: hydrodynamic phenomena (as punch flow, inertial forces or deterministic lateral displacement); hemodynamic phenomena (based on the intrinsic physical properties of the cells); and filters and physical filtration (based on micropores, microweirs, membranes and the gap between micropillars), as shown in Figure 5.

### 4.1. Hydrodynamic Separation and Sorting Techniques

**Figure 6:**
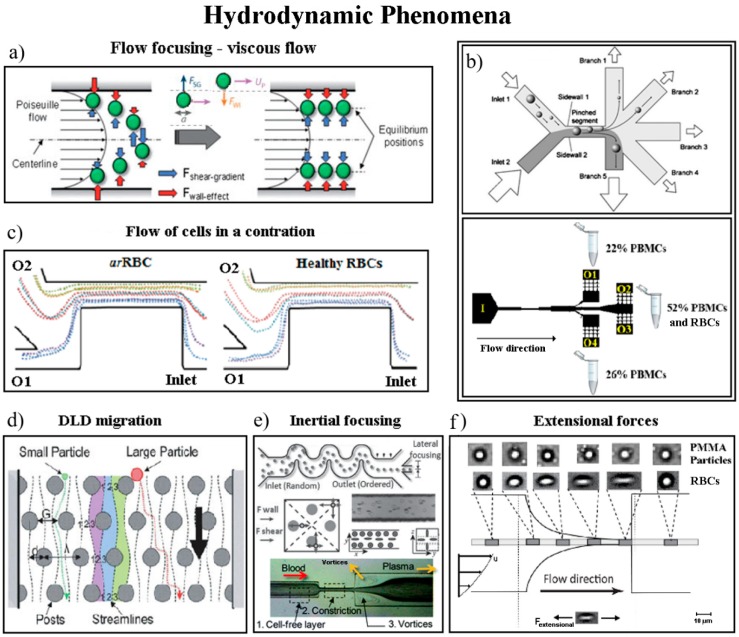
Hydrodynamic methods of separation: (**a**) the implied forces in a Poiseuille flow for cell separation. Reproduced with permission from [86] (**b**) the principle of hydrodynamic filtration in a microchannel with many outlets. Reproduced with permission from [81,84]. (**c**) trajectories analysis of rigid and deformable cells through a contraction for cell separation in two outlets. Reproduced with permission from [87]. (**d**) principle of deterministic lateral displacement. Reproduced with permission from [86]. (**e**) separation using inertial flow forces and at high flow rates creating vortices downstream a contraction. Reproduced with permission from [64,88]. (**f**) extensional forces for cell separation and mechanical analysis. Reproduced with permission from [89].

The hydrodynamic separation techniques are adequate for low Reynolds number flows (Re < 1) in the microfluidic devices. In a purely hydrodynamic flow separation technique, the laminar flow conditions exist, i.e., viscous forces are strong enough to have any disturbances in the pumped flow through the microchannel. In this process, the aligned cells are separated through multiple side branching outlets (Figure 6b), so that particles of different sizes will follow different paths, achieving size-based separation [83]. The hydrodynamic focusing is able to achieve narrow streams through sheath flows unlike the inertial focusing that occurs in a single flow stream.

Particles or cells exposed to a shear flow experience a lift force perpendicular to the flow direction and a force from the wall. The equilibrium of these two forces is responsible for the cells or particles migration and depends on several factors, such as channel geometry, flow rate, rheological properties of the carrier fluid and mechanical properties of the elements, as in Figure 6a. By manipulating the flow, for example, controlling the flow rate through one or more inlets, it is possible to achieve size-based cell separation and sorting [84].

The inertial separation methods generate the deflection of larger particles away from the flow, while smaller ones are carried on or near the original flow streamline. These mechanisms occur in curved and focused flow segments, and result from the combination of asymmetrical sheath flows and specific channel geometries, which are able to create a soft inertial force on the fluid. By using channels with curvature (as in Figure 6e), an additional drag force arising from secondary flows (called Dean vortices) enhances the speed of particle migration to more stable equilibrium positions, achieving a faster focusing of cells and particles than in straight channels, with high-throughput and continuous blood separation [64,70]. The inertial migration phenomenon has been widely recognised by the counteraction of two inertial effects, i.e., the shear gradient lift and the wall lift forces [85]. Many of the microfluidic devices have combined this separation inertial focusing strategy with other microfluidic methodologies to enhance blood cells separation, as different examples are presented in Figure 6.

Wang et al. [90], reported an inertial microfluidic device for continuous extraction of large particles or cells with high size-selectivity (under 2 µm) and high efficiency (above 90%). The authors developed a simple geometry with four key parts: a main microchannel, with a high-aspect-ratio geometry, to assure the inertial particle flow; two chambers for the formation of microvortexes, symmetrically positioned; two side outlets, positioned at the chambers’ corners, for the creation of sheath flow and removal of large particles; and, finally, an outlet for the small particles.

One of the hydrodynamic separation methods is based on the principle known as deterministic lateral displacement (DLD). This method employs arrays of pillars placed within a microchannel (array of obstacles). The laminar flow together with interactions in the array, forces the particles or cells to flow with specific trajectories through the device. The distance among the pillars is tailored according to the size of the cells or the particles to be sorted. The array pattern determines the displacement of cells or particles [84], i.e., the gap between posts and their offset determines the critical particle size for the fractionation. If the particles and/or cells are smaller than the critical size, they tend to flow through the array gaps without net displacement from the original central streamline. If the particles are bigger than the critical size, they will displace laterally, traveling at an angle predetermined by the posts offset distance (as shown in Figure 6d) [84]. Liu et al. [91] developed a rapid and label-free microfluidic structure for isolation of cancer cells from peripheral whole blood, using deterministic lateral displacement arrays (based on the size-dependent hydrodynamic forces), and achieved cells separation efficiency between 80% and 99% with a 2 mL/min throughput [91]. A high-throughput cytometry microsystem was reported by Rosenbluth et al. [92], to distinguish and quantify blood cell properties and help to prevent different hematologic problems (as sepsis, occlusion or leukastasis). The proposed microsystem presents a trifurcation into two bypass channels, and a network of bifurcations that split into 64 parallel capillary-like microchannels.

### 4.2. Hemodynamic Phenomena on Cell Separation Techniques

**Figure 7:**
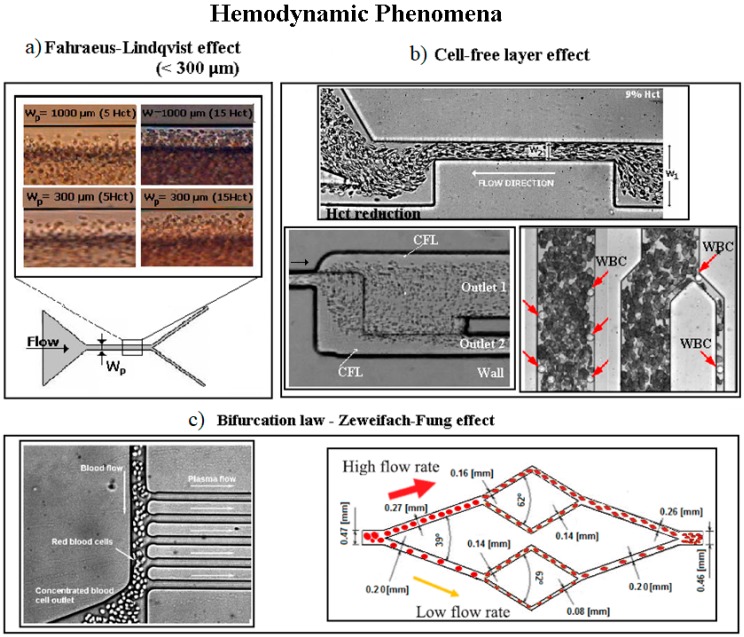
Blood separation microdevices based on hemodynamic flow separation techniques: (**a**) the Fåharaeus–Lindqvist effect in a microchannels with dimensions < 300 µm. Reproduced with permission from [23]. (**b**) cell-free layer as an advantage for cell and plasma separation and plasma skimming effect, WBCs margination. Adapted from [86,95,96]. (**c**) the Bifurcation law manipulated to remove cell-free plasma from blood and to mimic the microvasculature networks. Reproduced with permission from [86,97].

Microfluidic biomimetic cell separation techniques are based on mimicking the hemodynamic phenomena and the intrinsic properties of plasma and blood cells when flowing in microvessels. Different hemodynamic phenomena have been observed in vivo and replicated in microfluidic systems, including: plasma layer; Fåharaeus–Lindqvist effect (decrease of the apparent viscosity of blood in small vessels), which causes the tendency of the RBCs to migrate toward the centre of the microchannel, creating the cell-free layer (CFL) [11,93] (Figure 7b); leukocyte margination (migration of leukocytes, that are less deformable than RBCs, to the wall of the microchannel due to collisions between leucocytes and erythrocytes) [86]; plasma skimming (uneven distribution of red blood cells and plasma between the small side branch and the main channel); and the Zweifach–Fung bifurcation effect (in asymmetric bifurcations in which the vessel with the smaller flow rate gets a higher concentration of plasma), as represented in Figure 7c [64,93]. A number of microdevices have been developed to take advantage of these effects. For instance, in blood vessels with luminal diameter less than 300 µm, RBCs tend to migrate radially to the axial centre line of the vessel (Fåharaeus–Lindqvist effect), as shown in Figure 7a. Figure 7 summarizes the main hemodynamic phenomena of cell separation in microdevices. In microcirculation, the Zweifach–Fung bifurcation law is a relevant effect describing the cells tendency to travel to the daughter channel with a higher flow rate [86,88,94].

Jaggi et al. [94] developed a poly(methyl methacrylate) (PMMA) microdevice for blood plasma separation at high flow rates, based on the bifurcation law. The authors obtained, for Hct 4.5% and whole blood Hct of 45%, at a 5 mL/min^−1^ flow rate, separation efficiencies of 92% and 30%, respectively. The plasma yield obtained was 4% for the 45% Hct. The authors reported shear stress values much lower than the shear stress at which hemolysis occurs [94]. Lopes et al. [26] developed a microfluidic device able to perform separation of RBCs from plasma due to the cell-free layer (CFL) created upstream a contraction in a microchannel. The authors produced the device using a micromilling technique, and concluded that the geometric contraction produced by that technique was able to enhance the CFL, resulting in a low cost and efficient way to separate blood cells from plasma [26]. Faivre et al. [98] developed a microchannel with a constriction-expansion region for studying the Fahraeus effect, showing the increase of the cell-free region downstream of the constriction region. The authors collected almost pure plasma with Hct 16% at a flow rate of 200 μL·h^−1^, with a 24% yield (the separation efficiency was not mentioned explicitly). Lima et al. [99] successfully studied the behavior of RBCs in a 75 μm circular polydimethylsiloxane (PDMS) microchannel. The authors tracked individual RBCs (for 3% and 23% hematocrit) and observed that the trajectories of the solutions with higher RBC concentrations exhibit higher fluctuations in the direction normal to the flow. Additionally, the authors concluded that the RBCs flowing in a higher concentration environment tend to undergo multi-body collisions, increasing the amplitude of the RBCs’ lateral motion. Yang et al. [13] described a PDMS microfluidic device based on the Zweifach–Fung bifurcation law. The separation efficiency was defined in terms of hematocrit and quantified using an image processing program. The authors obtained, with the microdevice continuously running during 30 min without clogging, a separation efficiency of 100% for an inlet Hct of 45%, using defibrinated sheep blood, at a 10 μL·h^−1^ flow rate, with a yield or plasma volume percentage obtained of 15–25% [65]. During the last decade, Ishikawa et al., [67], Leble et al., [9] and Pinto et al., [23] have performed in vitro blood flow studies in simple microchannels with symmetric bifurcations and confluences and more recently Bento et al. [100,101] have performed similar studies in more complex geometries such as in microchannel networks. In those works, it was observed a clear cell-depleted layer at the region of the confluence apex that can be used to perform blood plasma separation.

### 4.3. Microfluidic Filters-Physical Filtration Techniques

**Figure 8:**
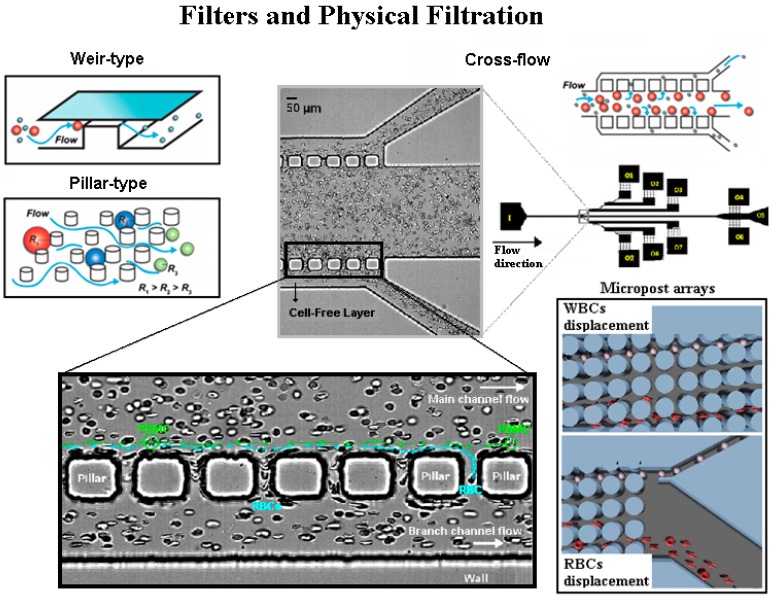
Schematic illustration for weir, pillar and cross-flow microfluidic filters. Images adapted from [81,84,109].

Combined with the mentioned separation techniques, microfluidic filters are usually introduced to increment the efficiency of the microfluidic devices [82,102]. Microscale filters, such as micropillar arrays, microweir structures or microporous membranes, are able to separate cells and particles based on their size and/or deformability. Although these filters allow the precise adjustment of the filter pore size to the required needs, they need to overcome different challenges, such as clogging of the microchannels, fouling and heterogeneity of the cell sizes [84]. Additionally, the design of the filters and barriers needs to take into consideration the different physical properties of the cells, including density, shape and deformability. Physical filtration microstructures, besides being a simple and non-destructive separation method, also allow the integration with other separation strategies. A major problem of the latter separation methodology is the high tendency to have clogging, jamming and possible blockage of the microdevice [103]. One way to minimize such a problem is by using cross-flow filters [83,104,105,106], since in cross-flow filtration, the fluid flows tangentially rather than through the filter as it does in membrane filtration (see Figure 8). This technique allows the particles to stay in a suspended state, avoiding their deposition, and can be used for separation of particles and cells. Crowley et al., [107] fabricated a passive crossflow filtration microdevice, operating entirely on capillary action, for the isolation of plasma from whole blood. Another method is by using micro-pillars that are suitably placed within the microchannel in a way that cells larger than the critical diameter follow a deterministic path while smaller cells maintain an average downward flow direction around the pillars, leading to the formation of multiple streams based size [86]. Chen et al., [105] developed a set of microfluidic chips based on the crossflow filtration principle, in which parallel micropillar-array and parallel microweirs were used to separate cells via their different sizes. Under the optimal conditions, more than 95% of the RBCs in a sample can be removed from the initial whole blood, while 27.4% of the white blood cells (WBCs) can be obtained. Plasma, WBCs and RBCs can be simultaneously separated and collected at different outlet ports with multilevel filtration barriers [105]. This principle is presented in Figure 8.

Zhang et al., [108] combined the use of hydrodynamic forces with passive filters comprised of artificial microbarriers of varying dimensions (that range in size from 15 to 7 μm, following the direction of fluid flow) in a chip to promote the flow and the separation of cells. By combining hydrodynamic forces with passive filters, the authors reported the separation of cancer cells based on their deformability. Additionally, by arranging the microbarriers in a rectangular, matrix-like structure, and by placing wide channels between post arrays, the authors ensured that the most flexible cells were able to seek alternate routes in the event of a blockage, as well as to regulate and equalize hydrodynamic pressure throughout the chip. The microscale geometry of the flow channels and post arrays ensured that the fluid flow is laminar, resulting in continuous cell movement and deformation in the device [108].

### 4.4. Comparison between the Separation Methods

Table 3 presents a comparison between the different categories of microfluidic cell separation, in terms of separation criteria, efficiency and throughput.

The referred passive separation methods are able to separate cells in a simple and non-destructive way and, furthermore, they allow easy integration of other processes in a single microdevice [114]. Ideally, a lab-on-a-chip platform should be small, simple and portable, by combining simple fluid driving mechanisms, reaction chambers and integrated detection systems for easy readout, making it able to be used by an end user or as a research tool, as a support for other laboratory technology [115].

Several authors have been approaching attempts for integration of passive separation of target cells and analysis, particularly of RBCs deformability assessment in a single microfluidic chip, which is still a challenge. Shevkoplyas et al. [116] developed a passive microfluidic device with a microvascular network perfusion system for cells separation and for measuring of the RBCs deformability. Faustino et al. [117] developed a microfluidic device in PDMS, with pillars and geometric variations, for the passive separation of RBCs, as well as to deform the cells and assess their deformability, by analyzing the acquired images. However, the proposed device is not fully integrated yet, since it still requires an external microscope for images evaluation and an external pumping system [117]. The described examples open new opportunities for research and show that lab-on-a-chip devices have high potential for integration of separation and detection tools in a single microfluidic platform.

There are still a lot of challenges to overcome regarding the integration of passive separation techniques in autonomous, functional and portable microdevices. Particularly, clogging, hematocrit, the amount of the sample and preparation time, mechanical stress (under relatively high pressure in microfluidic structures, several biological entities are at risk of rupturing, such as RBCs, or starting an adverse activation, such as platelets), contamination and biocompatibility are still challenging for the design and implementation of blood separation devices [64]. However, they also open new avenues for the miniaturization of analysis systems, requiring multidisciplinary synergies to assure the integration of microfluidics, actuation, detection, and readout systems in a single chip.

## 5. Perspectives

The research in the lab-on-a-chip area opens new possibilities for the miniaturization of analysis systems, requiring multidisciplinary synergies to assure the integration of microfluidics, actuation, detection, and readout systems in a single chip. All the developed efforts in this field are focused on the development of low-cost, portable, autonomous, multifunctional and commercial devices, with high sensitivity.

This paper presented an overview of the techniques used for separation of RBCs and the respective micro- and nanofabrication techniques, as well as examples of lab-on-a-chip devices with high potential for the integration of separation and detection tools in a single microfluidic platform. However, the use of these methods for separation of RBCs and detection of their properties has still a lot of challenges to overcome. Particularly, most separation methods, despite being able to separate particles, still need further development to be able to separate large cells, as RBCs, in microdevices, and require additional external equipment, which limits the methods’ portability.

As the lab-on-a-chip devices become multifunctional tools for separation and analysis of cells, without altering their state, efforts are converging into new analytical chemistry, diagnostic and treatment applications [118,119]. Further advances include lab-on-a-cell platforms [120,121] for isolation and individual characterization of cells and organ-on-a-chip devices for, among other applications, oxygenation studies [122,123], improvement of the clinical translation of nanomaterials for cancer theranostics, drug screening and personalized medicine [124,125].

## Figures and Tables

**Table 1:** Main advantages, disadvantages, resolution range and aspect ratio of microfabrication techniques used to develop microfluidic devices using polymer substrates. Adapted from [14].

Fabrication Technique	Advantages	Disadvantages	Resolution Range and Aspect Ratio
Hot embossing	Precise and rapid in the replication of microstructures. Mass production.	Restricted to thermoplastics. Time-consuming. Complex 3D structures are difficult to be fabricated.	Resolution between sub-100 nm and millimetre. Moderate aspect ratio (5:1) [32,33]
Injection molding	Mass production. Fine features. Low cycle time. Highly automated.	Restricted to thermoplastics. High cost mold. Nano-size precision is limited.	Resolution between sub-100 nm and millimetre. High aspect ratio (20:1) [34]
Laser photoablation	Rapid. Large format production.	Limited materials. Multiple treatment session. Difficulties for mass production. Micro-size precision is limited.	Resolution between micrometre and millimetre. High aspect ratio (30:1) [35,36]
X-ray lithography	High-resolution. Straight and smooth walls.	Complex and difficult master fabrication. Time consuming and high cost process.	Resolution between few nanometres and micrometres. Ultra-high aspect ratio (350:1) [37]
Soft-lithography	High-resolution and 3D geometries. Cost-effective. Excellent micro-size precision.	Pattern deformation and vulnerability to defects. Difficult to fabricate circular 3D geometries.	Resolution between 30 nm and 500 m. High aspect ratio (20:1) [18]
Xurography	Low-cost and rapid technique.	Complex 3D structures are difficult to be fabricated. Micro-size precision is limited.	Resolution between 150 m and millimetre. Moderate aspect ratio (8:1) [21,23,38,39]
Direct laser plotting	Low-cost and rapid technique. Free-mask technique. Good micro-size precision.	Complex 3D structures are difficult to be fabricated. Micro-size precision is limited. Reproducibility of the microdevices.	Resolution between 10–500 m. Moderate aspect ratio (7:1) [40,41]
Micromilling	Low-cost and rapid technique. Free-mask technique.	Complex 3D structures are difficult to be fabricated. Micro-size precision is limited. Reproducibility of the microdevices. Roughness.	Resolution between 30 m and millimetre. Moderate aspect ratio (8:1) [26,42]
Desktop fused deposition modeling (FDM), 3D-printing	Low-cost and rapid technique to fabricate prototypes.	Micro-size precision is limited. High roughness and complex to perform flow visualizations. Not suitable for mass production.	Resolution between 100 m and millimetre. Moderate aspect ratio (10:1) [43,44,45]
Nanofabrication	High-resolution of 2D and 3D geometries. Excellent nano-size precision. Highly repeatable, periodical structures.	High cost. Multiple process steps. Limited for microfluidic applications.	Resolution between 1–800 nm. Ultra-high aspect ratio (100:1) [17,46]

**Table 2:** Significant characteristics of the most common materials used for biomedical applications. Adapted from [51].

Characteristics	Silicon	Glass	Thermoplastics	Elastomers (PDMS)
Protein crystallization	Poor	Poor	Good	Moderate
Droplet formation	Excellent	Excellent	Good	Moderate
Porosity	Poor	Poor	Moderate	Moderate
Permeability	Poor	Poor	Moderate	Good
Bio-culture	Moderate	Moderate	Moderate	Good
Reusability	Yes	Yes	Yes	No
Disposable device use	Expensive	Expensive	Good	Good

**Table 3:** Comparison between the passive separation phenomena.

Method	Hydrodynamic Separation	Hemodynamic Separation	Physical Filtration
Separation criteria	Size	Size, deformability, cells concentration (hematocrit), cell aggregation [102]	Size, shape, deformability
Target sample	Cells, microparticles	RBCs, WBCs, plasma	Cells, particles
Separation Efficiency	Above 90% [90,110]; 80–99% [91]; 62.2% [111]	100% separation efficiency with 15–25% plasma separation volume [65]; 92% separation efficiency with diluted blood (Hct 4.5%) and 37% with whole blood (Hct 45%) [94]	More than 95% of the RBCs and 27% of the WBCs removed from whole blood [105]; 65–100% [102]; 98%, 8% (plasma from whole blood) [112,113]
Throughput	2 mL/min [91]; 10^6^ cells/min [110]; 1.2 mL/h (10^10^ cells/min) [111]	3–4 µL/min [112]; 5 mL/min [94]	2 × l0^3^ cells/s [112,113]
Potential effects on cells	Shear stress	Shear stress	Clogging, fouling, shear stress
Required instrumentation	Fluidic pumps	Fluidic pumps	Fluidic pumps
Processing layout	Continuous flow	Continuous flow	Batch; Continuous flow
